# Improving EEG classification of alcoholic and control subjects using DWT-CNN-BiGRU with various noise filtering techniques

**DOI:** 10.3389/fninf.2025.1618050

**Published:** 2025-08-19

**Authors:** Nidhi Patel, Jaiprakash Verma, Swati Jain

**Affiliations:** Department of Computer Science and Engineering, Nirma University, Ahmedabad, Gujarat, India

**Keywords:** alcoholism, deep learning, machine learning, noise filtering for EEG data, brain computer interface

## Abstract

Electroencephalogram (EEG) signal analysis plays a vital role in diagnosing and monitoring alcoholism, where accurate classification of individuals into alcoholic and control groups is essential. However, the inherent noise and complexity of EEG signals pose significant challenges. This study investigates the impact of three signal denoising techniques' Discrete Wavelet Transform(DWT), Discrete Fourier Transform(DFT), and Discrete Cosine Transform (DCT) Non EEG signal classification performance. The motivation behind this study is to identify the most effective preprocessing method for enhancing deep learning model performance in this domain. A novel DWT-CNN-BiGRU model is proposed, which leverages CNN layers for spatial feature extraction and BiGRU layers for capturing temporal dependencies. Experimental results show that the DWT-based approach, combined with standard scaling, achieves the highest accuracy of 94%, with a precision of 0.94, a recall of 0.95, and an F1-score of 0.94. Compared to the baseline DWT-CNN-BiLSTM model, the proposed method provides a modest yet meaningful improvement of approximately 17% in classification accuracy. These findings highlight the superiority of DWT as a preprocessing method and validate the proposed model's effectiveness for EEG-based classification, contributing to the development of more reliable medical diagnostic tools.

## 1 Introduction

Alcoholism is considered a significant health problem, causing a variety of physical, psychological, and social consequences (Manivannan et al., 2024). Detecting and monitoring the neurological effects of alcohol abuse is essential for prompt intervention, enabling effective treatment. EEG, a noninvasive technique that measures brain electrical activity, has emerged as a valuable tool to study the neural mechanisms associated with alcoholism (Rodrigues et al., 2019). However, analyzing EEG data presents notable challenges due to the presence of noise as well as artifacts, which can obscure meaningful brain signals. Therefore, implementing efficient noise filtering techniques is crucial to improve signal quality and improve the accuracy of subsequent analyses (Cohen et al., 2023).

Machine learning (ML), deep learning (DL), and statistical models have been widely adopted to classify individuals as alcoholic or control subjects based on EEG signals (Mumtaz et al., 2016; Faraz et al., 2024). EEG data, with its ability to capture neural activity patterns, serves as a reliable modality to identify alcoholism-related dysfunctions (Hosseini et al., 2021). These models utilize various features extracted from EEG signals, such as frequency bands, temporal dynamics, and connectivity measures, to achieve significant classification accuracy (Pain et al., 2023). Furthermore, EEG signals are widely used to detect and analyze emotional states, providing insight into the impact of addiction on emotional regulation (Akbari and Korani, 2023). The integration of advanced algorithms, including support vector machines (SVM), random forests, CNN, recurrent neural networks (RNN), and hybrid architectures such as CNN-LSTM-ATTN, further enhances the capability of interpreting complex, high-dimensional EEG data (Singhal et al., 2021; Siddiqa et al., 2024). Optimizing EEG electrode configuration has been shown to enhance classification performance and reduce complexity in neonatal studies (Siddiqa et al., 2025, 2023), a consideration also relevant for improving accuracy in addiction-related emotion recognition models.

Recent deep learning frameworks combining CNN-based feature extraction with fuzzy clustering have demonstrated effective classification of alcoholic and non-alcoholic EEG signals, showcasing robustness, noise resistance, and generalizability (Mei and Yi, 2024). In addition, statistical models help identify hidden trends and correlations, facilitating a deeper understanding of addiction and its emotional dynamics (Sanov et al., 2023). The multifaceted application of EEG in neuroscience research underscores its potential for diagnostic, therapeutic, and investigative purposes.

Given the susceptibility of EEG data to various sources of noise, such as muscle artifacts, eye blinks, and environmental interference, noise removal becomes a critical preprocessing step. Band-pass filtering is commonly applied to isolate frequency bands of interest while attenuating irrelevant signals (Mukhtar et al., 2021). Advanced techniques such as DWT, DFT (Roushdy et al., 2024), FFT (Fast Fourier Transform) (Cohen et al., 2023), and DCT (Discrete Cosine Transform) (Jyothirmy et al., 2023) have been widely employed to reduce non-stationary and time-varying noise by transforming signals into alternate domains where noise can be more effectively suppressed.

These methods not only improve the reliability of EEG signal analysis but also preserve the essential characteristics of neural activity. Techniques such as wavelet packet decomposition (WPD) and the use of specific wavelet families, such as Biorthogonal, have been shown to enhance classification accuracy, achieving performance levels as high as 99.87% (Rodrigues et al., 2019). Additional filtering techniques—such as high/low-pass filters, Second Order Blind Identification (SOBI), the ntzapline function, Common Average Referencing (CAR), and Laplacian filtering—further contribute to artifact removal and spatial resolution improvement (Sampedro-Piquero et al., 2024; Reddy and Sharma, 2024).

Despite these advancements, challenges remain. Many studies have yet to explore hybrid deep learning architectures that effectively capture both spatial and temporal EEG features (Farsi et al., 2021). Moreover, there is limited comparative research on how different noise filtering techniques—particularly DCT and DFT—impact the performance of deep learning models relative to DWT. There is a need for comprehensive studies that evaluate the combined effectiveness of noise filtering and deep learning techniques in accurately classifying alcoholic vs. control subjects (Li and Wu, 2022).

This article is structured as follows: Section 1 introduces the background and motivation for EEG-based classification in addiction research. Section 2 outlines the materials and methods used, including dataset description and preprocessing with DCT, DFT, and DWT. Section 3 presents the proposed approach, including a performance comparison of different models and pseudocode. Section 5 discusses the results and their implications. Section 6 concludes the paper with key findings and suggestions for future research.

### 1.1 Motivation

Alcohol addiction has a profound impact on brain function, and EEG serves as a reliable modality for detecting such neurological abnormalities. However, the classification of alcoholic brain signals is complicated due to high levels of noise and variability. Most existing studies rely on a single publicly available dataset and focus on limited preprocessing techniques. This highlights the need for a robust, noise-resilient deep learning framework that can improve classification accuracy and generalizability.

### 1.2 Key contributions

The main contributions of this work are as follows:

- A novel DWT-CNN-BiGRU model is proposed, which combines Discrete Wavelet Transform for noise filtering, Convolutional Neural Networks for spatial feature extraction, and Bidirectional Gated Recurrent Units for modeling temporal dependencies in EEG signals.
- A comparative analysis of three signal processing techniques—DWT, DFT, and DCT—is conducted to evaluate their effectiveness in EEG denoising for classification tasks.
- Standard scaling is applied before feature extraction to improve model convergence and accuracy.
- The proposed method achieves a classification accuracy of 94%, outperforming baseline models.
- A future direction is outlined to collect a new EEG dataset from clinically diagnosed alcoholic subjects to address the limitation of public dataset availability and enhance real-world applicability.

## 2 Materials and methods

### 2.1 Study design

This EEG data analysis study utilizes a publicly available dataset obtained from Kaggle.^1^ The dataset comprises EEG recordings from two categories of subjects: alcoholic and control individuals. The primary objective of the study is to apply various noise filtering techniques, implement an accurate deep learning model, and conduct a comparative analysis of different models to classify subjects effectively.

#### 2.1.1 Dataset

The dataset includes EEG recordings from 16 subjects, comprising eight alcoholic and eight control individuals. The data are acquired using 64 electrodes positioned according to standard sensor placements, with a sampling rate of 256 Hz. Each subject undergoes 30 trials, each lasting 1 second, enabling the analysis of brain responses under different stimulus conditions, including single stimulus (S1) and paired stimuli (S1 and S2). Each trial file contains various attributes such as trial number, sensor position, sample number, and sensor value—representing microvolt readings at each electrode location. Additional metadata, including subject identifiers (alcoholic or control), matching conditions, channel numbers, and timing information, provides essential inputs for preprocessing, noise filtering, and classification tasks using advanced machine learning models.

**Figure 1:**
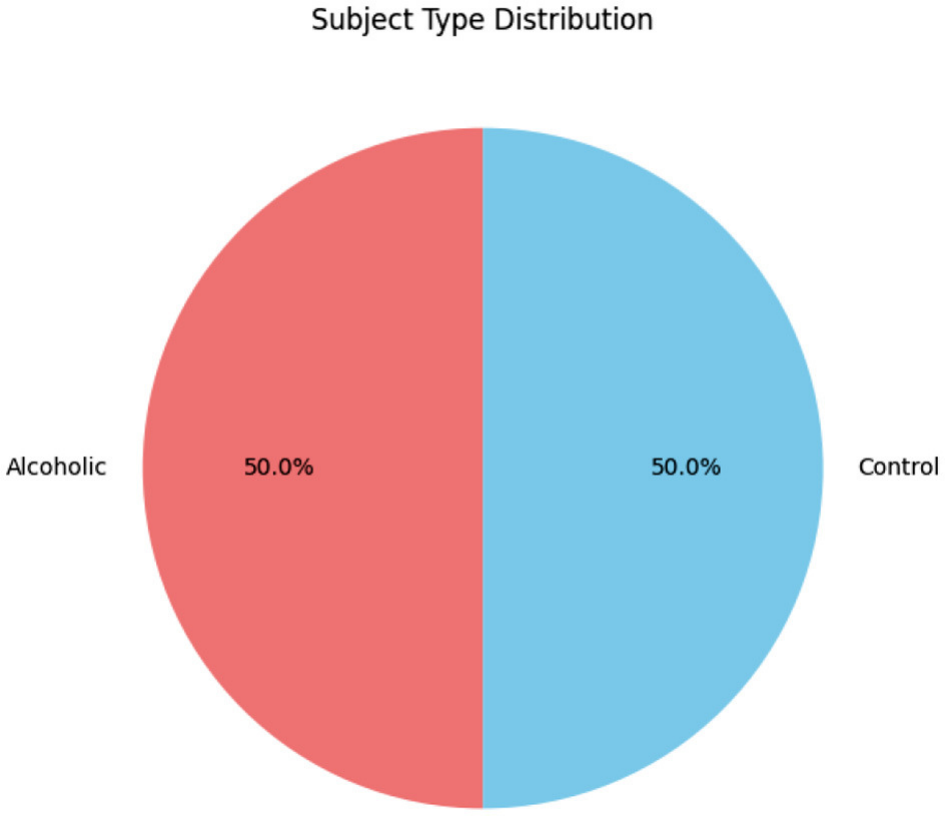
Distribution of alcoholic and control subjects in the dataset.

Figure 1 provides a visual summary of the subject distribution within the dataset. As shown in the pie chart, the dataset includes an equal number of alcoholic and control subjects in each category, ensuring a balanced representation for binary classification tasks.

### 2.2 Preprocessing

The preprocessing step involves standardizing the sensor position labels, transposing EEG samples, reorganizing the data, and restructuring the DataFrame to ensure consistency across trials as well as conditions. The data are then converted so that each row represents a sample from a single electrode. Subsequently, various noise filtering techniques' such as DCT, DWT, and DFT, are applied to clean the EEG signals. These noise-filtering methods are described in detail in the following sections.

### 2.3 Discrete cosine transform

The DCT transforms a discrete data sequence into a sum of cosine functions with varying amplitudes. Mathematically, the DCT of a sequence *x*[*n*], where *n* = 0, 1, …, *N*−1, is defined as:


$$X\left[k\right]=\overset{N-1}{\underset{n=0}{\sum }}x\left[n\right]\cos\;\left(\frac{\pi k\left(2n+1\right)}{2N}\right),\;k=0,1,\ldots ,N-1.$$

The noise filtering process using DCT involves three main steps:

1. Transform the noisy EEG signal from the time domain to the frequency domain using the DCT.
2. Identify and remove high-frequency components that correspond to noise, retaining the most significant lower-frequency coefficients.
3. Apply the IDCT(inverse DCT) to reconstruct the filtered signal in the time domain.

DCT is known for its efficiency in both noise reduction and signal compression, since it concentrates most of the signal's energy into a few significant coefficients. Unlike DFT, DCT uses only real numbers, which simplifies computations and reduces processing time. This characteristic makes DCT especially suitable for EEG signal processing, as it effectively separates meaningful neural activity from high-frequency noise (Mehla et al., 2023). However, selecting an appropriate coefficient threshold is critical to avoid losing essential signal information, and improper thresholding may introduce boundary artifacts.

#### 2.3.1 Discrete Fourier transform

The DFT converts a discrete time-domain signal into its frequency-domain representation, enabling the identification and removal of noise components. For a sequence *x*[*n*] of length *N*, the DFT is defined as:


$$X\left[k\right]=\overset{N-1}{\underset{n=0}{\sum }}x\left[n\right]e^{-j\frac{2\pi }{N}kn},\;k=0,1,\ldots ,N-1.$$

The DFT-based noise filtering process includes:

1. Transforming the noisy EEG signal into the frequency domain using the DFT.
2. Identifying frequency bands dominated by noise.
3. Designing and applying an appropriate filter (e.g., low-pass, high-pass, or band-pass) to attenuate unwanted components.
4. Applying the IDFT to reconstruct the cleaned signal in the time domain.

Although the DFT provides a precise frequency-domain analysis, it assumes signal stationarity and yields limited temporal resolution. Consequently, it may inadequately capture transient EEG events and requires careful filter design to preserve essential neural information.

#### 2.3.2 Discrete Wavelet transform

The DWT decomposes an EEG signal into wavelet coefficients, enabling efficient noise filtering and feature extraction. For a signal *X*(*t*), DWT expresses it as:


$$X\left(t\right)=\overset{J}{\underset{j=0}{\sum }}A_{j}\left(t\right)\phi_{j}\left(t\right)+\overset{J}{\underset{j=1}{\sum }}\overset{K_{j}}{\underset{k=1}{\sum }}D_{j,k}\left(t\right)\psi_{j,k}\left(t\right)$$

where *A*_*j*_(*t*) are approximation coefficients at scale *j*, ϕ_*j*_(*t*) is the scaling function, *D*_*j, k*_(*t*) are detail coefficients, and ψ_*j, k*_(*t*) are wavelet functions (Chandel et al., 2023).

We perform DWT-based noise filtering as follows:

1. We decompose the EEG signal into four levels, producing approximation coefficients *A*_4_ and detail coefficients *D*_1_, *D*_2_, *D*_3_, *D*_4_.
2. We apply soft thresholding to each detail coefficient *D*_*j*_, suppressing noise while preserving key neural components.
3. We reconstruct the denoised signal by inverting the DWT using the thresholded coefficients.

DWT captures transient and non-stationary features by isolating frequency-specific components of EEG signals, thereby enhancing our analysis of cognitive and emotional states. This approach improves the signal-to-noise ratio and supports more accurate tasks such as emotion recognition or alcoholism classification (Suryani et al., 2024; Li and Wu, 2022).

### 2.4 Classification

EEG signals classify subjects as alcoholic or control by identifying neural activity patterns associated with alcohol addiction and support clinical decision-making. Two hybrid deep learning models enable this classification: CNN-BiLSTM and CNN-BiGRU. Each model integrates CNN layers with bidirectional recurrent layers to capture spatial and temporal features from raw EEG signals. CNN layers apply one-dimensional filters to learn spatial dependencies across electrode channels, while bidirectional recurrent layers process sequential data in both forward and backward directions to capture temporal dynamics.

#### 2.4.1 CNN-BiLSTM

The CNN-BiLSTM model analyzes EEG data by combining spatial feature extraction with temporal sequence learning. It receives EEG input shaped as (time steps, channels) through an input layer. Three 1D convolutional layers with 64, 128, and 128 filters apply ReLU activation to extract spatial features. Each convolutional layer follows a MaxPooling1D layer to reduce dimensionality. Dropout layers with a rate of 0.5 follow selected layers to prevent overfitting. Two bidirectional LSTM layers process the pooled features, with the first layer returning sequences to preserve temporal structure. Dropout layers follow each LSTM layer to enhance generalization. A fully connected dense layer with softmax activation produces classification probabilities over the encoded labels. The model compiles with the Adam optimizer at a learning rate of 0.001 and uses sparse categorical cross-entropy loss.

#### 2.4.2 CNN-BiGRU

The CNN-BiGRU model adopts the same convolutional architecture as CNN-BiLSTM and replaces LSTM layers with GRU layers. It applies three 1D convolutional layers with ReLU activation, each followed by MaxPooling1D and dropout layers. Two bidirectional GRU layers then capture temporal dependencies while reducing computational complexity. Dropout layers follow each GRU layer. A final dense layer with softmax activation generates classification probabilities. The model is compiled with the Adam optimizer, using a learning rate of 0.001, and optimizes sparse categorical cross-entropy loss.

**Figure 2:**
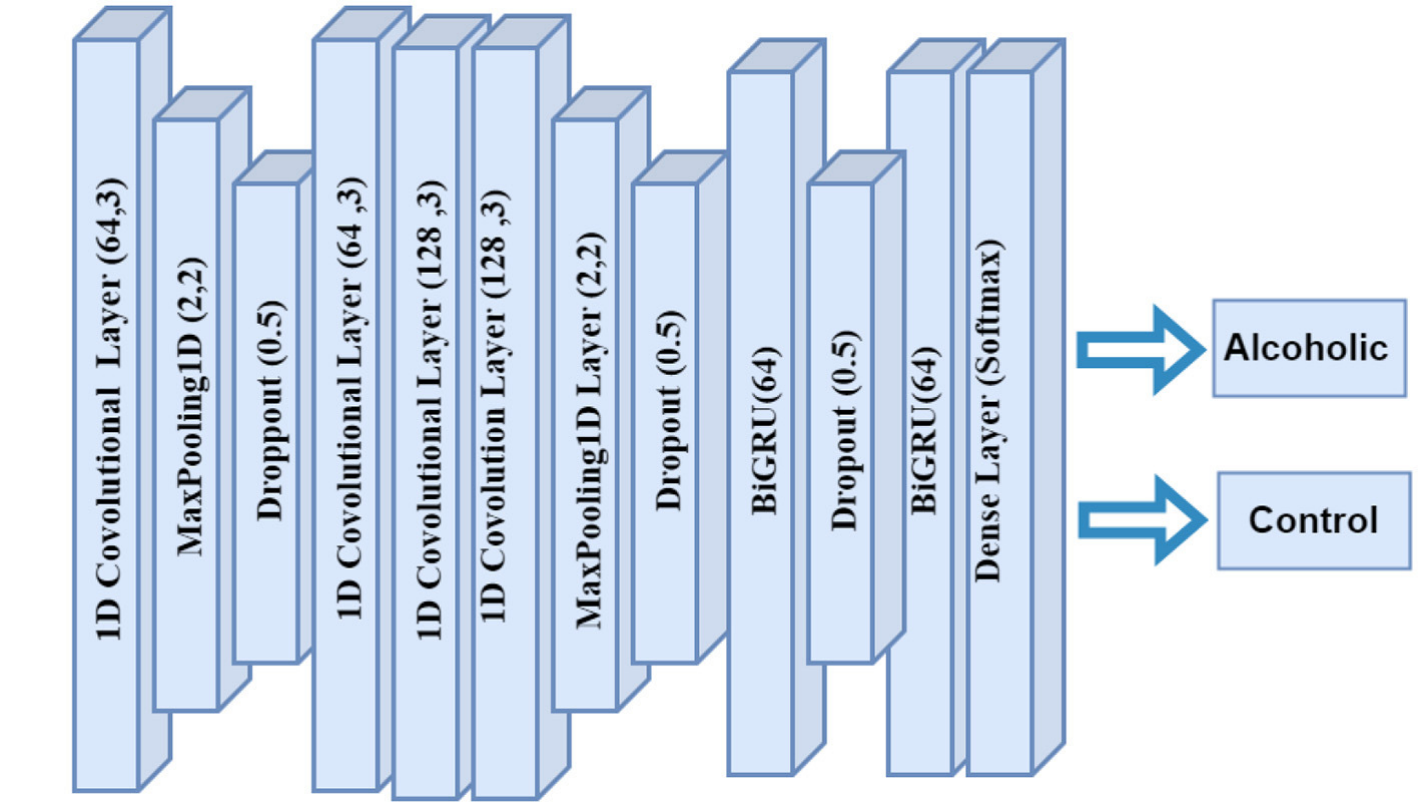
CNN-BiGRU layer architecture.

The CNN-BiGRU model architecture integrates CNN with Bi-GRUs to analyze EEG data effectively. In Figure 2, the model takes input data structured with a specified shape, followed by a series of Conv1D layers that extract spatial features through convolution, utilizing 64 and 128 filters with a kernel size of 3. Each convolutional layer is accompanied by MaxPooling1D layers to downsample the data and reduce computational load, while dropout layers with a 0.5 rate mitigate overfitting by randomly disabling neurons during training. The architecture then incorporates two bidirectional GRU layers, which capture temporal dependencies in both forward and backward directions, enhancing the model's ability to learn complex patterns in the data. Finally, a dense output layer applies a softmax activation function for multi-class classification, facilitating the identification of various emotional states or neurological conditions based on the EEG signals. The model is compiled with the Adam optimizer and sparse categorical cross-entropy loss function to optimize performance during training.

## 3 Proposed research

**Figure 3:**
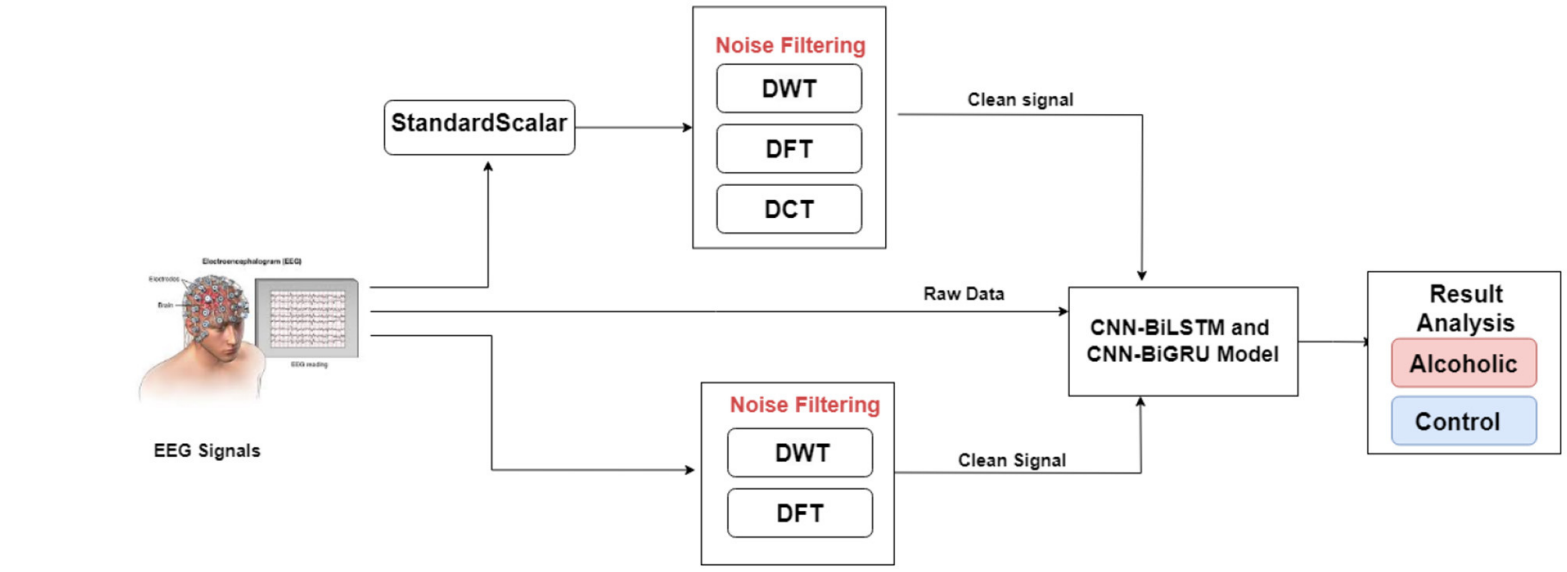
Proposed research.

Figure 3 illustrates the experimental architecture. This study applies various noise filtering techniques to EEG data to improve signal quality before classification by deep learning models. The preprocessing pipeline uses the Standard Scaler to normalize features by subtracting the mean and scaling to unit variance, thereby enhancing model performance.

The study employs DCT, DWT, and DFT to remove noise. These methods eliminate unwanted components to produce cleaner input data, which proves crucial for accurate classification.

After preprocessing, the filtered EEG data enter the CNN-BiLSTM and CNN-BiGRU models for classification of subjects as alcoholic or control. By integrating these noise filtering techniques, the proposed research aims to enhance deep learning model accuracy and overall classification performance.

To optimize the model's performance, we conducted hyperparameter tuning by testing various batch sizes (32, 64, 128, and 200) and epoch settings (50 and 100 epochs). The Adam optimizer with a learning rate of 0.001 was used throughout all experiments. After comparison, the best results were obtained using a batch size of 200 and 100 epochs, especially when used in combination with the DWT preprocessing and standard scaling. Stratified 5-fold cross-validation was applied to ensure robustness and fairness across the alcoholic and control groups. This cross-validation strategy helped evaluate the model's consistency and avoid overfitting on specific subjects.

### 3.1 Comparison analysis

The study evaluates the impact of applying standard scaling, along with the effectiveness of various noise filtering techniques–namely DFT, DWT, and DCT, on classification performance. Data processed with and without standard scaling is input into both CNN-BiLSTM and CNN-BiGRU models to classify subjects as either alcoholic or control. Standard scaling, by normalizing feature values, enhances model learning and improves overall performance, as reflected in the evaluation metrics.

**Figure 4:**
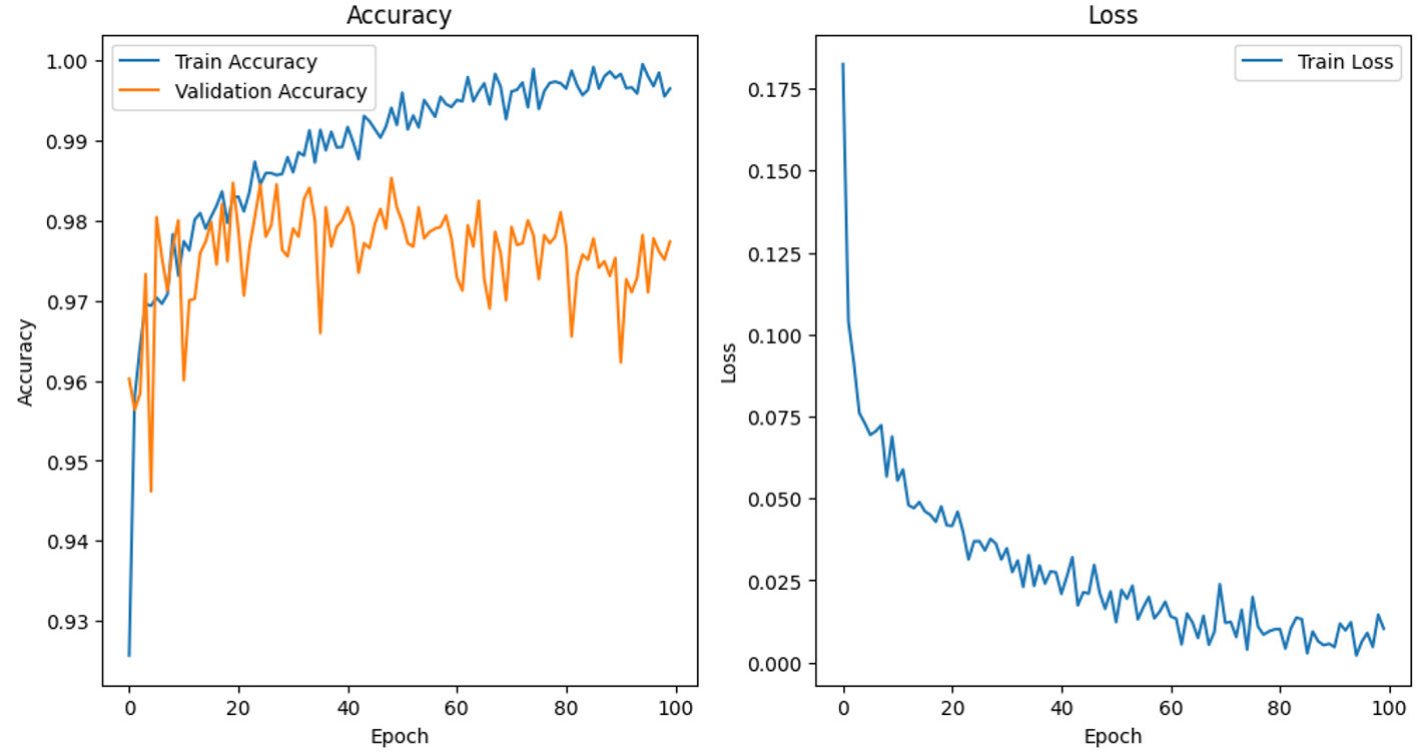
Results of accuracy.

Figure 4 Illustrates the accuracy vs. epoch for the model's performance, with the number of epochs set to 100 during evaluation. This graph provides insight into how each model converges and stabilizes in accuracy over time, highlighting the effectiveness of different filtering and scaling configurations. The comparison of noise filtering techniques across both models is summarized in a table, including accuracy, precision, recall, and F1 score as key performance metrics. To ensure the robustness and generalizability of the models, 5-fold cross-validation was employed. The average performance metrics across all folds are reported, offering a reliable estimate of model stability under varying data splits. This analysis serves as a guide for optimal preprocessing strategies in EEG data classification using deep learning models.

**CNN-BiLSTM**: This model performed well, particularly when combined with Discrete Wavelet Transform (DWT) and standard scaling, achieving 93% accuracy. The CNN layers extract spatial features from the multichannel EEG input, while the BiLSTM captures sequential dependencies.

**CNN-BiGRU**: This model yielded the highest accuracy (94%), suggesting that GRU's simpler architecture and fewer parameters contribute to more efficient learning without compromising performance. BiGRU captures bidirectional temporal features more effectively in this case, possibly due to better generalization on small datasets.

## 4 Discussion

The ability of the suggested DWT-CNN-BiGRU model to effectively extract and use both spatial and temporal characteristics from EEG signals is responsible for its superior performance. Denoising the EEG data while maintaining crucial time-frequency components makes the Discrete Wavelet Transform (DWT) an effective preprocessing method. This raises the quality of the input data that is fed into the model and increases the signal-to-noise ratio. While the Bidirectional Gated Recurrent Unit (BiGRU) records both past and future temporal relationships across time steps, convolutional neural networks (CNNs) are very good at extracting spatial patterns across the 64 EEG channels. These elements work together to create a hybrid architecture that improves classification accuracy by learning comprehensive EEG representations.

In this study, the CNN-BiGRU model was designed to effectively process and classify EEG signals by capturing both spatial and temporal dependencies. The input EEG data were first reshaped to a 3D format of (samples, timesteps, 1) and standardized using StandardScaler to normalize the amplitude values across all electrodes and trials. The architecture includes multiple 1D convolutional layers for spatial feature extraction, followed by max pooling and dropout layers to reduce overfitting. Two bidirectional GRU layers (each with 64 units) were added to learn the temporal sequence information from both directions of the EEG signal. The final dense layer with softmax activation outputs the classification into alcoholic or control classes.

Performance results from different preprocessing and model configurations are summarized in Table 1, with the DWT + StandardScaler + CNN-BiGRU configuration demonstrating superior classification accuracy.

**Table 1:** Performance metrics for different noise filtering techniques for various models.

**Noise filtering technique**	**Model**	**Accuracy**	**Precision**	**Recall**	**F1 score**
Raw data	CNN-BiLSTM	90%	0.90	0.91	0.91
DFT	CNN-BiLSTM	68.94%	0.58	1.00	0.73
DFT with standard scalar	CNN-BiLSTM	78%	0.58	0.99	0.73
DCT	CNN-BiLSTM	70%	0.64	0.93	0.76
DWT with standard scalar	CNN-BiLSTM	93%	0.93	0.96	0.95
DWT with standard scalar	CNN-BiGRU	94%	0.94	0.95	0.94

Alcoholic brain signals are known to exhibit distinct patterns compared to non-alcoholic or control subjects. Neurological studies have shown that alcohol abuse leads to alterations in brain wave activity, particularly affecting the alpha, beta, and theta frequency bands. These changes manifest as variations in amplitude, signal coherence, and latency, which are captured in EEG recordings. In this study, visual differences were analyzed through distribution plots and classification accuracy metrics. Additionally, techniques such as feature importance ranking and attention visualization can be integrated in future work to support explainability and interpretability of the model. Such explainability studies can help to highlight which electrodes or time segments contribute most to the classification decision, offering insight into brain regions affected by alcoholism.

Although the proposed model demonstrates strong performance, there remain areas for further optimization. The reliance on a single public dataset limits the generalizability of the results. A key enhancement would involve collecting a larger and more diverse EEG dataset from clinically diagnosed alcoholic patients, which would allow the model to learn from more varied brain patterns. Additionally, model complexity can be reduced through architecture pruning or lightweight deep learning frameworks, making the model suitable for real-time or portable EEG devices. Another potential enhancement lies in combining multiple noise filtering techniques (e.g., hybrid DWT-DFT) or incorporating attention mechanisms to focus on the most informative time steps or channels.

Compared to existing techniques, the proposed DWT-CNN-BiGRU model exhibits superior performance in processing and classifying EEG signals related to alcoholism. The integration of DWT enables better noise filtering and signal decomposition, while CNN and BiGRU components provide complementary spatial and temporal feature extraction. This combination achieves higher accuracy, precision, recall, and F1-score than baseline models such as CNN-BiLSTM or those using only DFT/DCT.

The proposed model demonstrates several advantages, including enhanced feature extraction through convolutional layers, reduced overfitting achieved by dropout and standardization, and effective sequence modeling via recurrent layers. These components collectively contributed to strong classification performance on EEG data. Nonetheless, the model's reliance on a relatively small dataset and its substantial computational requirements present notable limitations. To address these challenges, future work may focus on incorporating subject-specific adaptations, as EEG signals exhibit considerable variability across individuals. Techniques such as personalized calibration or transfer learning could improve model generalizability. Furthermore, exploring advanced architectures, including attention mechanisms and transformer-based models, holds potential for capturing more complex temporal dependencies and further improving classification performance.

This study utilizes a publicly available EEG dataset from Kaggle, which is widely used in previous research due to the limited availability of open-access datasets related to alcohol-addicted individuals. While this dataset provides a useful foundation for evaluating the proposed method, relying on a single dataset may reduce the diversity of results. To overcome this limitation, future work will involve collecting and analyzing a new EEG dataset from clinically diagnosed alcohol-dependent subjects. This will help strengthen the validation and improve the real-world relevance of the proposed approach.

## 5 Conclusion

This study focused on the challenging task of classifying individuals as alcoholics or controls using EEG signals, an essential step toward advancing early diagnosis, treatment monitoring, and therapy planning for addiction-related disorders. A hybrid CNN-BiGRU model integrated with Discrete Wavelet Transform (DWT) was proposed to enhance the extraction of both spatial and temporal features from EEG data. Comparative evaluation with other filtering methods, including DFT and DCT, demonstrated that the DWT-CNN-BiGRU model outperformed the widely used DWT-CNN-BiLSTM model, achieving higher classification accuracy. The application of standard scaling further improved model performance by reducing feature variability. The proposed framework contributes a robust and effective approach for EEG-based classification in addiction research. While the results are promising, future work should explore hybrid noise filtering techniques, larger and more diverse datasets, and multiclass classification schemes to improve the model's generalizability and clinical relevance.

## Data Availability

The original contributions presented in the study are included in the article/supplementary material, further inquiries can be directed to the corresponding authors.

## Footnotes

^1^EEG-Alcohol – kaggle.com. Available online at: https://www.kaggle.com/datasets/nnair25/Alcoholics. (Accessed November 28, 2024).
